# Microbial Community Characteristics Largely Unaffected by X-Ray Computed Tomography of Sediment Cores

**DOI:** 10.3389/fmicb.2021.584676

**Published:** 2021-04-12

**Authors:** Erica Ewton, Scott Klasek, Erin Peck, Jason Wiest, Frederick Colwell

**Affiliations:** ^1^Department of Microbiology, Oregon State University, Corvallis, OR, United States; ^2^College of Earth, Ocean, and Atmospheric Sciences, Oregon State University, Corvallis, OR, United States; ^3^Department of Veterinary Medicine, Oregon State University, Corvallis, OR, United States

**Keywords:** microbial communities, computed tomography scanning, storage, sediment, biomarker, geological cores

## Abstract

X-ray computed tomography (CT) scanning is used to study the physical characteristics of soil and sediment cores, allowing scientists to analyze stratigraphy without destroying core integrity. Microbiologists often work with geologists to understand the microbial properties in such cores; however, we do not know whether CT scanning alters microbial DNA such that DNA sequencing, a common method of community characterization, changes as a result of X-ray exposure. Our objective was to determine whether CT scanning affects the estimates of the composition of microbial communities that exist in cores. Sediment cores were extracted from a salt marsh and then submitted for CT scanning. We observed a minimal effect of CT scanning on microbial community composition in the sediment cores either when the cores were examined shortly after recovery from the field or after the cores had been stored for several weeks. In contrast, properties such as sediment layer and marsh location did affect microbial community structure. While we observed that CT scanning did not alter microbial community composition as a whole, we identified a few amplicon sequence variants (13 out of 7,037) that showed differential abundance patterns between scanned and unscanned samples among paired sample sets. Our overall conclusion is that the CT-scanning conditions typically used to obtain images for geological core characterization do not significantly alter microbial community structure. We stress that minimizing core exposure to X-rays is important if cores are to be studied for biological properties. Future investigations might consider variables, such as the length and energy of radiation exposure, the volume of the core, or the degree, to which microbial communities are stressed as important factors in assessing the impact of X-rays on microbes in geological cores.

## Introduction

Geological cores are used to study Earth’s layers and chemistry, and X-ray computed tomography (CT) scanning is often used to acquire images and conduct analyses of such cores (Orsi et al., 1994). X-ray CT scanning provides non-destructive, high-resolution, 3D views of sediment structure and macroscopic constituents (Davey et al., 2011). This technique has been used by geologists for decades (Petrovic et al., 1982) to provide data about lithology, particle size, and micropaleontology, allowing inference about sediment accumulation rates, chronological development of sediments, or to assist in the reconstruction of past climate conditions (Ericson et al., 1961; Poore et al., 2005).

In the last 30 years, numerous studies of the shallow and deep Earth biosphere show that microbial life is widely distributed in geological systems (Edwards et al., 2012; Colwell and D’Hondt, 2013). Accordingly, more investigations are conducted jointly between microbiologists and geologists to gain insight into biological communities that exist in geological materials and the biologically-induced processes that may be evident in such cores. X-ray CT scanning has been used to observe biomineralization (Benzerara et al., 2004), aspects of formation structure, such as porosity and pore shape, as they relate to microbial colonization (Nunan et al., 2006), and the presence of fractures in sediments that may advect fluids that support microbial communities (Yao et al., 2019) or to understand microbial contributions to pore-scale soil architecture (Crawford et al., 2012).

Under certain conditions, X-rays are destructive to living cells. Numerous studies have been conducted on the effects of X-rays on mammalian cells due to the value and common use of X-rays in diagnostic applications in humans and animals (Webb, 2017). In a study aimed at understanding how eukaryotic cells might be influenced by low doses of X-rays, yeast cells were found to be sensitive to persistent low levels of X-ray exposure (Mercier et al., 2004). With respect to microorganisms, one area of research deals with the degree to which different microbial cells are susceptible to sterilization by X-rays (Morrissey and Phillips, 1993). X-ray radiation can cause double-stranded breaks in microbial DNA (Dean et al., 1969), and applications have been developed for using X-rays to sterilize surfaces, albeit at high levels of the ionizing radiation (Borgognoni et al., 2017). Radiation effects on polymers include cross-linking and further polymerization as well as the formation of free radicals and peroxides (Kleland et al., 1993). Ionizations predominantly occur in aqueous conditions and the reactive compounds are able to cause single- and double-strand breaks in cellular DNA depending on the radiation dose (Russell, 1993).

The effect that CT scanning has on microbial functionality and microbial community composition within sediment cores has received some attention but results vary among different studies. In one case, CT scanning caused an immediate decrease in β-glucosidase and dehydrogenase activity; however, enzyme levels, measures of microbial functional properties, returned to normal 21 days after X-ray exposure (Bouckaert et al., 2013). A different study found slightly lower potential extracellular enzyme activity of β-glucosidase, chitinase, and phosphatase in scanned vs. unscanned cores after approximately 3 weeks and more DNA in scanned vs. unscanned cores, suggesting that CT scanning increased DNA availability (Fischer et al., 2013). This study also found a difference in community structure between scanned and unscanned samples; however, the molecular technique they used did not allow high resolution of the microbial community members. Additionally, their X-ray exposure times were considerably longer (2 h) than that typically used to conduct a geological scan. Another study found no change in the levels of microbial biomass present in a soil core following repeated CT scanning but did not consider other properties of microbial communities (Zappala et al., 2013). The degree to which microbial communities are altered by this important geological technique remains unresolved, leading us to an examination of how and whether key taxa change as a result of X-ray exposure.

The objective of this study was to determine whether X-ray CT scanning alters microbial community diversity and composition in sediment cores. This was accomplished by comparing 16S rDNA sequence analysis of microbial community diversity (using high-throughput sequencing to obtain amplicon sequence variants, or ASVs) in subsamples from scanned and unscanned replicate cores immediately after scanning and for several weeks thereafter. We sampled and compared three sediment layers at two sites from a tidal saline wetland across a range of depths and organic matter (OM) content values. By examining sediment materials with a range of characteristics, we aimed to assess how microbial communities inherent to different subsurface conditions and core structures might respond differently to the CT scanning treatment. Findings from this study suggest that X-ray CT scanning with doses typically used in geological investigations largely does not alter microbial community structure for the sediment samples that we examined, though a minor subset of taxa showed differential relative abundances that may have important considerations when analyzing certain populations from scanned sediment cores.

## Materials and Methods

### Site Description and Sample Recovery

To examine the effect of X-ray CT scanning on microbial communities in sediments, duplicate 1.5 m-long, 10 cm-diameter geological cores were collected on July 5, 2018 from two areas in the Netarts Bay high marsh on the Oregon coast (45.373778, −123.96489 and 45.372197, −123.964223, with elevations 2.772 m and 2.765 m, respectively, Supplementary Figure S1) as previously described (Peck et al., 2020). Air and sediment temperatures were not measured at the time of sampling; however, Netarts Bay water temperatures have been reported to range between 10 and 13°C in July (Barton et al., 2012). The Northern site is more proximal to the estuarine waterline (Supplementary Figure S1), therefore allowing greater tidal influence and resulting in increased water and nutrient fluxes. The dominant vegetation types in both coring areas at the time of collection were *Deschampsia cespitosa* (tufted hairgrass) and *Juncus balticus* (Baltic rush; Peck et al., 2020). These locations, defined as tidal saline high marsh/scrub-shrub wetlands, were chosen because Peck et al. described easily accessible short cores with variable OM content. The duplicate cores from each site – one to be CT-scanned and one as an unscanned control – were split lengthwise to reveal sedimentary features, including a distinct sand layer of several cm thickness. This layer is interpreted as having been deposited by a tsunami in 1700 based on stratigraphy and depth (Darienzo and Peterson, 1990; Shennan et al., 1998). We focused on three sediment horizons taken from above, within, and below the sand layer, respectively, termed as “Shallow,” “Middle,” and “Deep,” thus increasing the diversity of geological materials (and presumably microbial communities) to be evaluated. Shallow and Deep sediment layers were silty and high in OM (8.2–31% by weight), whereas the denser sand layers contained 2.2–5.2% (Supplementary Table S1).

### Experimental Design, X-Ray CT Scanning, and Core Analysis

After splitting, one half-core from each site was scanned using a Toshiba Aquilion 64-slice CT unit (parameters: 120 kV peak, 400 mA, 0.5 pitch, 200 mAS, 41.0 HP, and 0.641 pitch factor) at Oregon State University’s Veterinary Hospital. These parameters, including the voltage used, are consistent with those commonly used to achieve high resolution images of geological cores (Reilly et al., 2017). Supplementary Figure S2 shows a section of a scanned core with a clearly visible sand layer. The dose delivered to each half core was 66.6 mGy, which was consistent with doses used to scan geological cores (Zappala et al., 2013). After scanning, half cores were covered in plastic wrap to minimize oxygen exposure and dehydration, then stored at 14°C in the dark to maintain approximate *in situ* conditions. Within hours, the surface-most few mm of the half cores became red apparently due to oxidation of reduced iron. Sediment appeared not to lose water content throughout the duration of this study, as no condensation accrued on the plastic wrap and sediment remained consistent textures. On days 0, 7, 14, and 21 after coring, the surface 1 cm of sediment was removed, then three subsamples were taken from the inner sediment in each layer of each half core. Depths of subsampling in the Shallow, Middle, and Deep layers for the Northern site were 53–56, 60–63, and 69–72 cm, respectively, and 54–57, 59.5–61.5, and 64–67 cm, respectively, for the Southern site. Additional subsamples were taken from shallow, middle, and deep layers in each core and freeze-dried for 2 days at the OSU Core Lab to eliminate water. Freeze-dried samples were later examined for organic carbon weight percentage using standard loss on ignition protocols (Heiri et al., 2001).

### DNA Extraction, Purification, Amplification, and Sequencing

DNA from each subsample was extracted using a MoBio PowerSoil DNA Isolation Kit following manufacturer’s instructions (QIAGEN Inc., Germantown, MD; Marotz et al., 2017). A purified water sample was used as a sediment-free extraction control. Bacterial and archaeal 16S rRNA genes were amplified in triplicate following the Earth Microbiome Protocol (Caporaso, 2018), using 515-forward and 806-reverse universal primers targeting the V4 hypervariable region (Caporaso et al., 2011). Primers also contained dual-indexed Illumina sequencing adaptors (Kozich et al., 2013). Amplification was verified with gel electrophoresis, and pooled amplicons were purified using a QIAQuick PCR Purification kit according to the manufacturer’s instructions (QIAGEN Inc.). DNA concentrations were quantified using a QuBit dsDNA High Sensitivity Kit as per manufacturer’s instructions. Illumina MiSeq V2 paired-end 250 bp sequencing was performed at the Center for Genomic Research and Bioinformatics (CGRB) at Oregon State University.

### Analysis of Sequence Data

16S rRNA gene sequence data were processed in R version 3.6.1 using DADA2 version 1.12.1 (Callahan et al., 2016a) to filter sequences, to define amplicon sequence variants (ASVs), and to remove chimeras according to an established workflow (Callahan et al., 2016b). Version 132 of the SILVA nonredundant 16S reference database (Quast et al., 2013) was used to assign taxonomies to ASVs. Sequences were aligned with DECIPHER version 2.12.0 (Wright et al., 2012), and phylogenetic trees were generated with Phangorn 2.5.5 (Schliep, 2011). Phyloseq version 1.28.0 (McMurdie and Holmes, 2013) was used to combine sample information, count and taxonomy tables, and phylogenetic trees into a single object for subsequent analyses in R. Sequences identified as eukaryotes, chloroplasts, mitochondria, or belonging to unclassified domains were removed from both datasets. Using the prevalence-based method within decontam version 1.4.0 (Davis et al., 2018), we identified and removed one contaminant ASV (a member of *Methanolobus*) that was more abundant in one extraction blank than other samples. In addition to the extraction blank, 10 samples that contained fewer reads than the blank were pruned from the phyloseq object, leaving 44 remaining samples with ≥4,622 reads.

Alpha diversity calculations (observed richness and Shannon index) were calculated within Phyloseq. ASV read tables were transformed using hellinger and cumulative sum scaling approaches using vegan version 2.5-6 (Oksanen et al., 2008) and metagenomeSeq version 1.26.3 (Paulson et al., 2013), respectively. Vegan was also used to calculate and ordinate distance matrices and implement PERMANOVA tests among sediment layers, and DESeq2 version 1.24.0 (Love et al., 2014) was used to determine biomarker ASVs based on scanned/unscanned treatments using adjusted *p* ≤ 0.05.

## Results

The objective of this study was to determine whether microbial community structure in geological materials is altered by CT scanning, an analytical technique commonly used for non-destructive characterization of cores. Altogether, 16S rRNA libraries from 44 sediment samples collected from two coring sites in Netarts, OR, United States (Supplementary Figure S1) across a range of depths, sediment layers, and storage times passed our sequence processing pipeline and contained a total of 7,037 Bacterial and Archaeal ASVs. Of these, 22 samples (11 pairs of scanned/unscanned treatments) otherwise had virtually the same metadata and were sampled no more than 2 cm depth apart from replicate cores.

**Figure 1:**
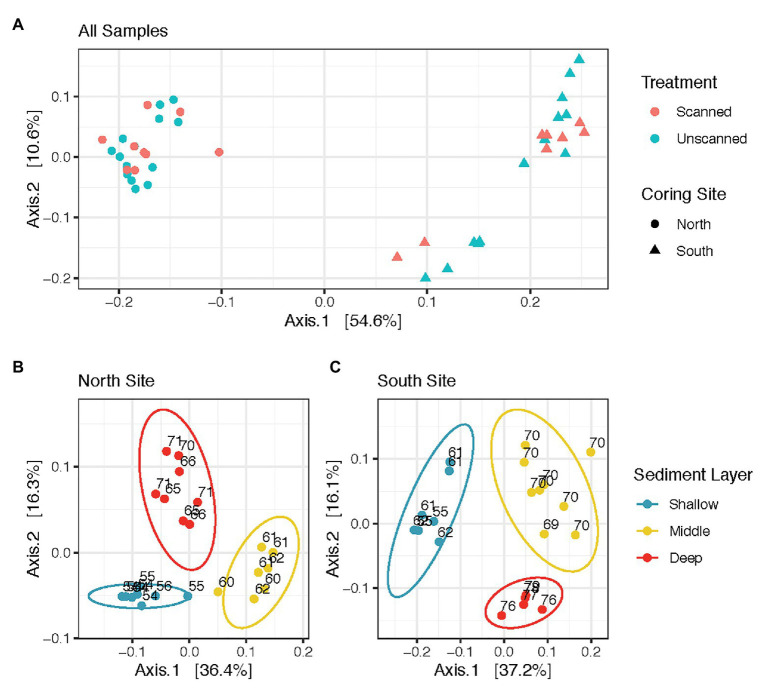
Sediment microbial community structure does not vary as an effect of CT-scanning, but varies between coring sites and layers within the sediment column. **(A)** displays all communities analyzed in this study, while **(B,C)** show those from North and South sites, respectively. All panels are Principal Coordinate Analysis (PCoA) ordinations of weighted Unifrac distances calculated from Cumulative Sum Scaling-transformed read counts, with each axis showing its contribution to the variation among communities analyzed.

Microbial communities from all spatial and temporal subsamples of scanned and unscanned cores from North and South coring sites are shown in the ordination (Figure 1A). PERMANOVA tests comparing all scanned and unscanned communities revealed that scanning was only able to explain 1.2–2.4% of community variation and did not affect community structure overall (values of *p =* 0.347 and 0.74, using Binary Jaccard and weighted Unifrac distances, respectively). These observations were robust to the transformation methods used on the read count data (Cumulative Sum Scaling and Hellinger). When unpaired samples were discarded from the tests, 0.6–2.0% more variation in communities could be attributed to scanned/unscanned treatment.

Communities showed differences in composition according to sample site (Figure 1A). PERMANOVA tests comparing communities in North vs. South coring sites yielded significant differences (*p* < 0.001, with up to 51% of variation attributed to site). Within communities from North and South coring sites, the three different sediment layers also revealed differences (*p* < 0.001, with up to 47% variation based on layer, Figures 1B,C). However, communities from North and South sites were unaffected by storage times of up to 22 days at 14°C (*p* > 0.5 for each site, with less than 4% variation based on storage time, Supplementary Figure S3).

**Figure 2:**
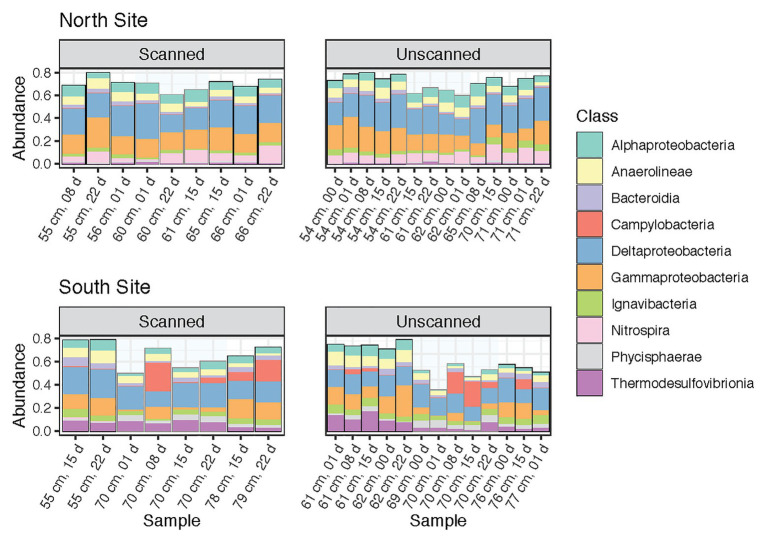
Community composition barplots showing the 10 most abundant classes (all within Bacteria) within all samples analyzed, sectioned by site (North and South) and CT-scanning treatment. These classes contain 68.4% of reads within the dataset. Samples are ordered by increasing depth, and then by storage time.

This study was designed to test microbial community changes in a range of sediment samples, including multiple coring sites, sediment layers and depths, and times of storage following coring and CT scanning. Percent abundances of dominant classes (those containing >2% of reads across all samples) among paired subsamples are shown in Figure 2. These 10 bacterial classes comprised 68.4% of the reads in the dataset. Their proportions remained consistent between scanned and unscanned samples, suggesting that their abundances were not altered by X-ray CT treatment of the cores. Instead, the composition of the most abundant classes reflects the high degree to which coring sites explain community differences (Figure 1A). In particular, Nitrospira, known to be broadly distributed nitrite-oxidizers (Lücker et al., 2010), were more abundant in communities from the North site, whereas Thermodesulfovibrionia were more abundant in the South.

Proportions of the most abundant classes were remarkably consistent throughout storage, though in tsunami deposit layers from the South site, Campylobacteria increased after 8 days and later decreased after 15 or 22 days (Figure 2). Slight but significant differences were seen when comparing sediment layers within each core. Gammaproteobacteria dominated nearly all communities (along with Deltaproteobacteria), but was noticeably less abundant in Southern tsunami deposit samples. In addition, Zixibacteria, a bacterial phylum previously known as RGB-1 and considered to be metabolically diverse (Castelle et al., 2013), accounted for 18.6% of reads in Southern tsunami deposit samples as compared to 5.0% of the reads in samples from above or below the sand layer (*p* = 2.8e-5). This difference was not detected across Northern samples, where Zixibacteria comprised 0.5% of communities on average.

We found that CT scanning did not affect microbial community alpha diversity for samples obtained from either coring site or for any of the sediment layers. CT scanning did not significantly alter observed ASV richness or Shannon indices among all paired (scanned vs. unscanned) samples, yielding respective *t*-test values of *p* 0.248 and 0.118. Neither did these alpha diversity metrics change as sediment cores were stored for up to 22 days (Supplementary Figure S4). Depth was the only factor that contributed to community alpha diversity, showing positive correlations with both metrics tested for only South samples (*t*-test values of *p =* 0.009 for observed richness and 0.017 for Shannon).

**Figure 3:**
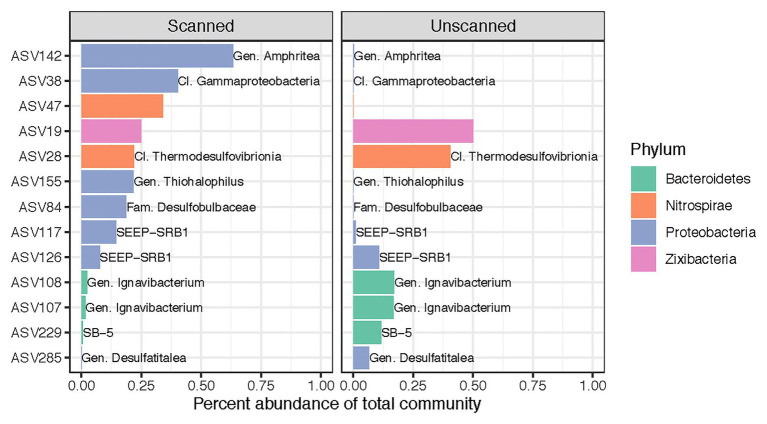
Differentially abundant ASVs across all 22 sets of paired scanned/unscanned samples. Mean percent abundances of these 13 ASVs across each treatment are plotted and colored according to their phylum. Labels, where present, show the most specific taxonomic category or clade to which each ASV belongs.

Despite our finding that CT scanning did not alter microbial community composition as a whole, we identified 13 ASVs (out of 7,037) that showed differential abundance patterns between scanned and unscanned samples among paired sample sets (Figure 3). These biomarkers for scanned or unscanned treatments included seven Proteobacteria, three Bacteroidetes, two Nitrospirae, and one Zixibacterium. Two of these ASVs include members of the SEEP-SRB1 clade, sulfate-reducing Deltaproteobacteria often observed in association with anaerobic methanotrophic archaea (Schreiber et al., 2010; Kleindienst et al., 2012). Two ASVs belonging to the genus Ignavibacterium, which contains metabolically versatile, facultatively anaerobic, chemoheterotrophic, and potentially mixotrophic members (Liu et al., 2012), showed lower abundances in scanned cores, though 25 other ASVs from this genus were present in the dataset and not detected as biomarkers.

While we found evidence that CT scanning altered the observable microbial communities in the samples only slightly, sediment layers and coring sites were important factors in determining the microbial community structure in the samples that we examined. Coring sites and sediment layers differed in levels of OM and dry bulk density (DBD), presumably providing habitats for different organisms with different nutrient needs (Supplementary Table S1). OM in the northern core was approximately 10, 3, and 9% for above, within, and below the sandy tsunami deposit, respectively. OM in the Southern cores was higher: approximately 17, 5, and 31% for above, within, and below the tsunami deposit, respectively. Our DBD and OM trends followed an inverse relationship, consistent with previously collected data from the same sites (Peck et al., 2020).

## Discussion

In our study, we examined the effect of CT-scanning on microbial communities in cores at the highest resolution yet attempted (i.e., to the level of ASVs), which enabled us to determine the degree to which community structure might be changed by the X-ray analytical method. Differential abundances of several ASVs among scanned and unscanned communities may imply variation in stress tolerance mechanisms at the species level and is consistent with variable dynamics within sulfur-cycling Desulfocapsa (Finster et al., 2013) OTUs in long-term ex situ marine sediment enrichments (Klasek et al., 2019). Our finding that several ASVs may be sensitive to scanning suggests that those interested in studying these populations may instead opt to sample unscanned replicate cores when available or sample before scanning. However, because we found no genus whose ASVs were all affected, putative responses to scanning may be variable at species or subspecies levels.

Our study is similar to previous investigations on the effects of X-ray CT scanning on soil microbes. In one investigation, using phospholipid fatty acid signatures to detect changes in microbial community structure, no difference was found among microbial communities that could be attributed to X-ray exposure (Bouckaert et al., 2013). However, in contrast with our findings, (Fischer et al., 2013) observed a difference in microbial community diversity in scanned vs. unscanned soils immediately after low levels of sustained irradiation, indicating that microbial assemblages were changed by the X-ray analysis. Subsamples taken from outer layers of their microcosms showed a more pronounced change in community structure than subsamples taken from the inside of the microcosms, suggesting that the soils may have shielded microbes from radiation exposure. That Fisher et al. noted differences in community responses to CT scanning compared to our study may be due to several factors. Their experiments were conducted on sieved soils that were established as microcosms and then incubated for 2 weeks prior to scanning. In contrast, we used intact (minimally disturbed) core sediments and scans were performed shortly after collection. Consequently, their microbial communities were subject to considerable additional manipulation and incubation prior to scanning and sample collection compared to our samples, which are more representative of freshly collected cores.

In addition to community changes, Fischer et al. (2013) noted an increase in retrievable DNA (as noted by DNA amplicon levels) in scanned cores compared to unscanned cores, which they considered to be a possible indicator of more dead or dying organisms in the former. We did not measure a difference in the amount of retrievable DNA in the respective samples of our study but we cannot discount the possibility that our analysis may have included free DNA. Our methods were not designed to differentiate between free or cell-associated DNA, although such methods have been published (Patel et al., 2012; Alawi et al., 2014). Dead cells can contain amplifiable DNA for up to 3 weeks (Josephson et al., 1993) and extracellular DNA can be stable for months to years in sediments under anoxic conditions (Dell’Anno and Corinaldesi, 2004; Torti et al., 2015). If CT scanning damages or kills cells in sediment cores without also making the DNA unextractable or unamplifiable, then activity- or cultivation-based investigations would be adversely affected. Future studies should assess how CT scanning may cause cell damage or lysis, whether treatment increases the extracellular DNA pool after extraction, and the degree to which microbial activities may change.

We observed no significant effect of storage time on the communities in both scanned and unscanned cores, which suggests that scanning under these conditions may not be an influential factor in determining microbial community structure over timescales of a few weeks, at least for the samples that we studied. Others have detected changes in subsurface microbial community structure as a result of long-term storage between the time that cores are collected and when microbiological analyses are conducted (Brockman et al., 1998; Mills et al., 2012). Our results notwithstanding, microbiological samples should be stored at −80°C to minimize shifts in abundance of some microbes in the samples unless selection of certain microbes is a deliberate goal.

Our findings, and those of similar studies (e.g., Zappala et al., 2013), can be used to select CT-scanning methods when imaging geological cores that will also be studied for microbiological properties. Our results indicate that the X-ray CT scanning parameters we used to capture images of the sediment cores from this estuary did not notably alter the microbial communities, with the possible exception of a few taxa. Thus, we recommend future studies implement conditions similar to those we used (e.g., 120 kV peak, 400 mA, 0.5 pitch, 200 mAS, 41.0 HP, 0.641 pitch factor, calculated to deliver a dose of approximately 60–70 mGy) to avoid alteration of microbial community structure within sediment cores while still achieving adequate image quality for examining the physical properties of cores. This X-ray dose is among the lowest of the values reported by Zappala et al. in their review of multiple scanning studies.

We may consider a number of additional studies to examine the details of microbial survival in X-ray CT-scanning. For example, a study designed to determine the threshold at which communities change as X-ray dose is increased and how they change, which would identify sensitive taxa at progressively higher doses. Determining the effects of sample size on communities in a scanned core may also be important. A large diameter core or large sample volume may effectively shield cells located in the center of the cores as opposed to those on the outside (Fischer et al., 2013). Geomicrobiologists often use subcores by collecting samples from the center of a core to avoid contamination from drilling fluids, the core barrel, or any smeared material from the core barrel (Griffin et al., 1997). For CT-scanned cores, subcoring may be beneficial as these samples may be most shielded from X-rays. Water content (measured *via* DBD) of a core may also be important as radiation can cause the formation of free radicals and peroxides, which may be harmful to a variety of cellular macromolecules such as DNA, proteins, and lipids (Kleland et al., 1993). It would also be valuable to determine how cellular physiological status (e.g., actively growing vs. dormant cells) affects survival after scanning.

We suspect that other factors played a role in differentiating microbial communities found at the respective study sites. For example, the Northern coring site is located closer to estuarine waters and therefore may be more tidally influenced, which could determine differences in macroflora, the flux of water and nutrients in the system (Supplementary Figure S1), and ultimately the microbial flora. Sediment composition or source of sediments may influence microbial community structure as different sediments can have distinctive nutrient concentrations (Kachi and Hirose, 1983) and grain sizes (DeFlaun and Mayer, 1983). Regardless of the environmental characteristics of the cores at the different sites and how these might control the microbial members, the microbial communities that we sampled provided a heterogeneous assemblage of cells to submit to CT testing.

Overall, our observations suggest that CT scanning did not alter the microbial communities or relative abundances of taxa when using DNA extraction and sequencing methods commonly used by microbiologists. This suggests that CT scanning to discern key geological or sedimentological features can be conducted without significantly altering the structure of the microbial community as measured using DNA extraction and gene amplification and sequencing protocols. It is important for geologists and microbiologists to coordinate while studying the same core material to assure that samples are maintained under conditions acceptable to both scientific disciplines during analysis.

## Data Availability Statement

The datasets presented in this study can be found in online repositories. The names of the repository/repositories and accession number(s) can be found at: https://www.ncbi.nlm.nih.gov/, PRJNA533633.

## Author Contributions

EE and FC designed the study. EE, SK, EP, JW, and FC collected the samples, conducted the experiments, and wrote the manuscript. EE, SK, and FC analyzed the data. All authors contributed to the article and approved the submitted version.

### Disclaimer

This report was prepared as an account of work sponsored by an agency of the United States Government. Neither the United States Government nor any agency thereof, nor any of their employees, makes any warranty, express or implied, or assumes any legal liability or responsibility for the accuracy, completeness, or usefulness of any information, apparatus, product, or process disclosed, or represents that its use would not infringe on privately owned rights. Reference herein to any specific commercial product, process, or service by trade name, trademark, manufacturer, or otherwise does not necessarily constitute or imply its endorsement, recommendation, or favoring by the United States Government or any agency thereof. The views and opinions of authors expressed herein do not necessarily state or reflect those of the United States Government or any agency thereof.

### Conflict of Interest

The authors declare that the research was conducted in the absence of any commercial or financial relationships that could be construed as a potential conflict of interest.
