# Supplementary material for: Microbial Community Characteristics Largely Unaffected by X-Ray Computed Tomography of Sediment Cores
Source: Front Microbiol. 2021 Apr 12;12:584676. doi: 10.3389/fmicb.2021.584676 (PMC8072469; doi:10.3389/fmicb.2021.584676)
Supplement: Supplementary file 1 [file Image_1.pdf]

Supplementary Material

1 Supplementary Figures

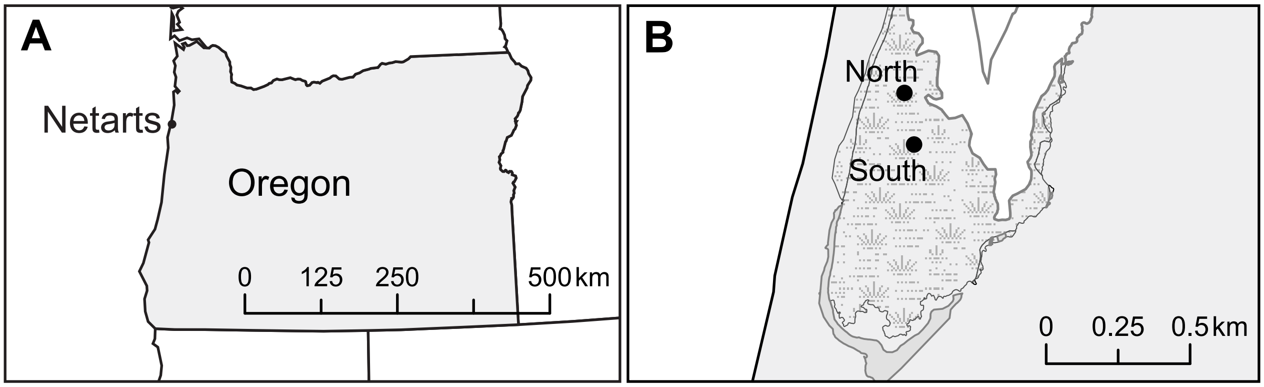

Figure S1. Map showing location of study area and coring sites in Netarts, OR, USA. The map of Netarts (B) was created using the Pacific Marine & Estuarine Fish Habitat Partnership's West Coast, USA Estuarine Biotic Habitat maps (Brophy et al., 2019). Vegetated salt marsh is displayed as a light gray marsh pattern.

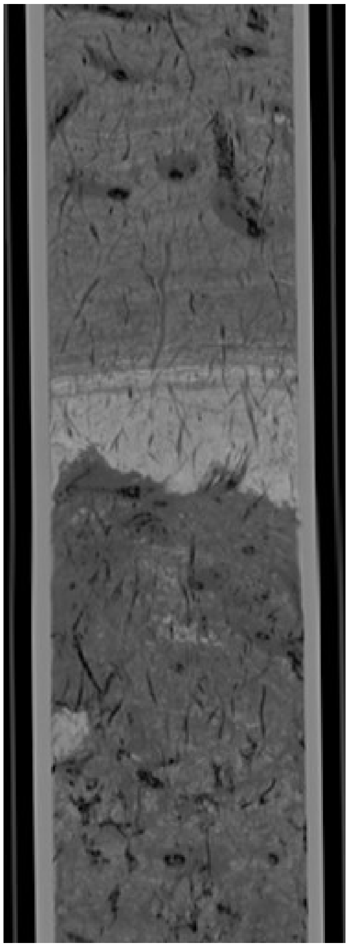

Figure S2. CT-scanned image of core subsection. This image shows approximately 40 cm of one sediment core (10 cm diameter), oriented as originally collected with the top of the image corresponding to the shallower portion of the subsection. The light-colored area in the middle of the image is the “middle” layer tsunami deposit that was 60-64 cm deep in the original core.

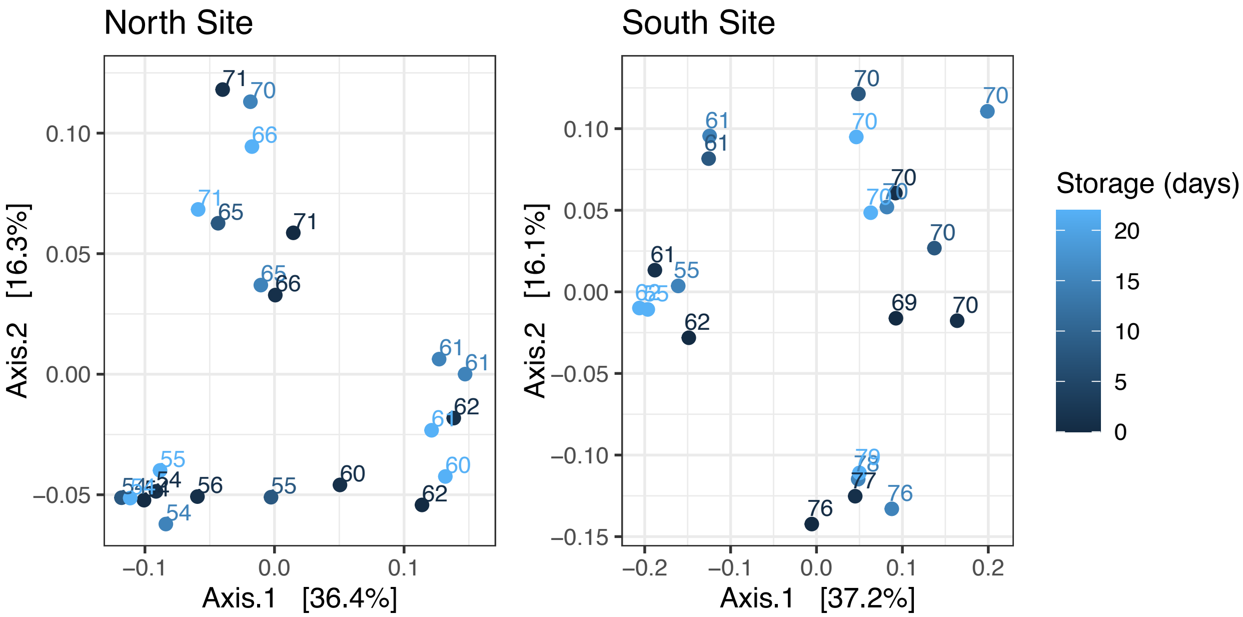

Figure S3. Sample storage time does not correlate with microbial community structure. PCoA ordinations are the same as in Figures 1B and 1C, but colored by the number of days samples were stored in dark at 14˚C. Ordinations show weighted Unifrac distances calculated from Cumulative Sum Scaling-transformed read counts, with each axis showing its contribution to the variation among communities analyzed.

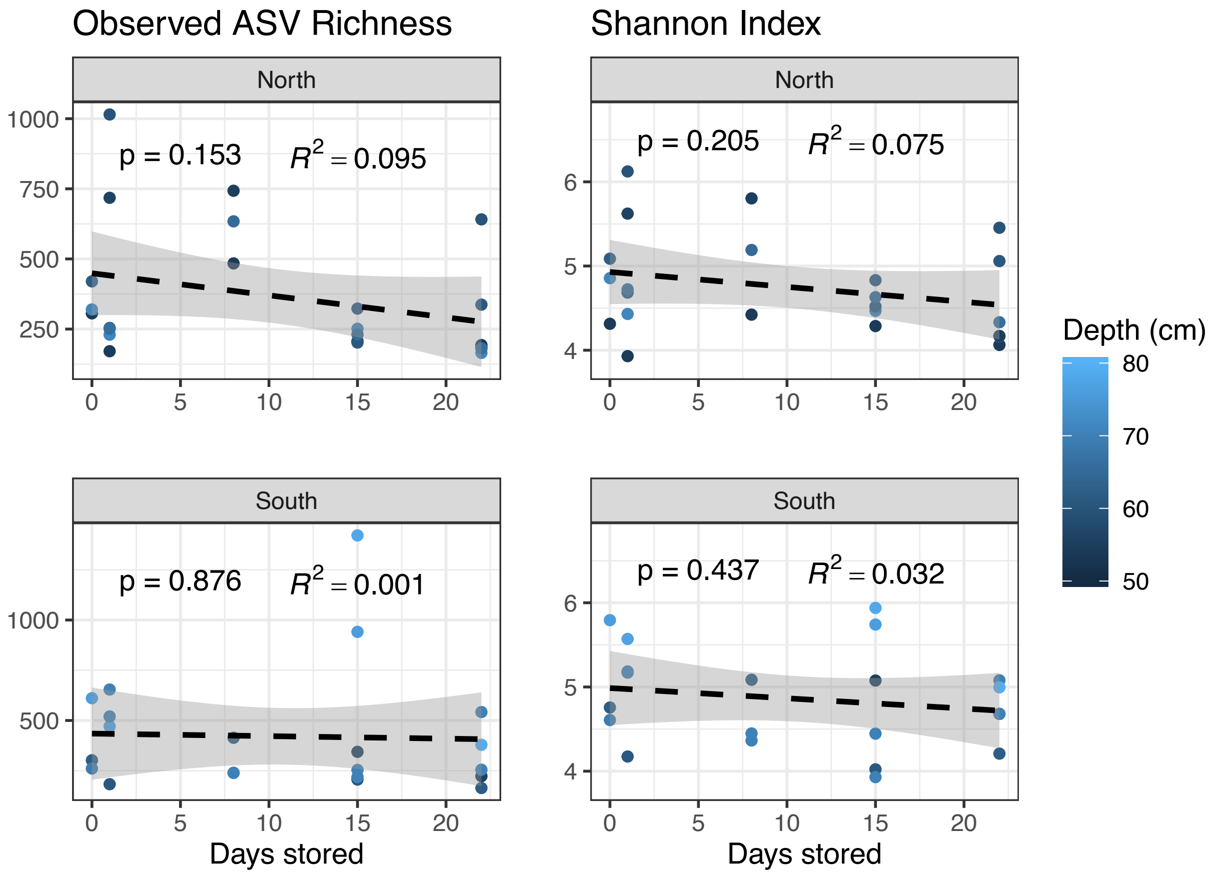

Figure S4. Two metrics of alpha diversity plotted over storage time for all samples, separated by North and South coring sites. Points are colored by depths within the sediment column. Linear regression p-values and multiple R^2 values show that alpha diversity does not significantly change with storage time.

2 Supplementary Table

Coring Site (sediment layer)	Depth (cm)	OM (%)	DBD (g/cm^3)
North (Shallow)	54	9.36	0.91
**North (Shallow)**	**54**	**11.72**	**0.88**
North (Middle)	62	2.20	0.98
**North (Middle)**	**60**	**4.13**	**0.96**
North (Deep)	69	8.18	0.92
**North (Deep)**	**65**	**9.03**	**0.91**
South (Shallow)	62	15.71	0.84
**South (Shallow)**	**55**	**19.05**	**0.81**
South (Middle)	70	5.17	0.95
**South (Middle)**	**70**	**5.24**	**0.95**
South (Deep)	76	29.88	0.70
**South (Deep)**	**78**	**31.72**	**0.68**

Table S1: Measurements of organic matter (OM, percent) and dry bulk density (DBD, g/cm^3) for each sediment layer. CT-scanned samples are bolded and placed below their paired unscanned samples. OM and DBD show opposing relationships in sand layers, as lowest OM and highest DBD are measured in the "Middle" samples when compared to surrounding "Shallow" and "Deep" layers.

Supplemental Reference

Brophy, L. S., Greene, C. M., Hare, V. C., Holycross, B., Lanier, A., Heady, W. N., et al. (2019). Insights into estuary habitat loss in the western United States using a new method for mapping maximum extent of tidal wetlands. *PLOS ONE* 14, e0218558. doi:10.1371/journal.pone.0218558.
